# Experiences of school health professionals in implementing structured assessments of sexual health and experiences of violence among youth in Sweden using the SEXual health Identification Tool (SEXIT): a qualitative sequential study

**DOI:** 10.1136/bmjph-2024-001667

**Published:** 2024-11-26

**Authors:** Miranda Håkansson, Sanny Söderström, Marlene Makenzius

**Affiliations:** 1 Department of Health Sciences, Mid Sweden University, Östersund, Sweden

**Keywords:** Adolescent, Health Personnel, School Health Services, Sexual Health, Violence

## Abstract

**Introduction:**

Poor sexual and reproductive health (SRH) is a global public health concern, particularly among adolescents. While school health services (SHS) play a crucial role in preventative care, professionals are inconsistent in addressing SRH issues. This study explored school health professionals’ (SHPs) experiences of an implementation of structured assessments of sexual health and experiences of violence among youth in Sweden using the SEXual health Identification Tool (SEXIT).

**Methods:**

A qualitative sequential study was conducted from October 2023 to January 2024 to explore the experiences of 57 SHPs trained in the SEXIT method. Data collection included a questionnaire with open-ended questions, group discussions and individual interviews, analysed using qualitative content analysis.

**Results:**

Addressing sexual risk taking and violence was considered a priority to help youths make informed SRH choices. SEXIT aided SHPs in acting as educators to promote SRH and freedom from violence, normalised conversations about sensitive topics, facilitated the identification of students needing support, and could be integrated as a routine part of preventive work within SHS. However, hindering factors for effective implementation included a lack of supportive leadership and a unified approach among involved organisations to clarify roles, responsibilities and referral pathways. Furthermore, it is essential to further adapt SEXIT and train SHPs to meet the specific needs of vulnerable youths and perpetrators, ensuring equitable support.

**Conclusions:**

SEXIT offers unique opportunities for SHPs to discuss SRH and violence with school youths, a priority part of their preventive work; however, it is not routinely used, underscoring the need for supportive leadership and a unified approach among organisations.

**WHAT IS ALREADY KNOWN ON THIS TOPIC**

- The SEXual health Identification Tool (SEXIT) is an assessment tool designed to identify youth with experiences of sexual risk taking and violence, developed for use in youth-friendly clinics. The implementation of SEXIT within school health services (SHS) in Sweden has just begun (2021–2024) and has not yet been evaluated.

**WHAT THIS STUDY ADDS**

- The findings demonstrate that SEXIT is an asset within SHS and can be routinely integrated as part of preventive work, as it provides a platform for educating and discussing sensitive topics, which is a priority for school health professionals. However, vulnerable groups and perpetrators of violence require further attention. For effective and equitable implementation, SEXIT needs supportive leadership and a unified approach among organisations to clarify roles, responsibilities and referral pathways.

**HOW THIS STUDY MIGHT AFFECT RESEARCH, PRACTICE OR POLICY**

- Further research is needed in the use of SEXIT with youth who have limited understanding of the Swedish language and/or intellectual disabilities, as well as with those who are perpetrators of violence. The study highlights the need for tailored interventions and policies to ensure equity in support for all youths, providing valuable insights for the continued implementation of SEXIT within SHS.

## Introduction

 Poor sexual and reproductive health (SRH) is a global public health issue, particularly for adolescents aged 10–19.^1^ Adolescence is critical for establishing healthy sexual and reproductive behaviours and addressing harmful issues such as inequitable gender norms and gender-based violence.^2^ Violence includes intimate partner violence (IPV) and non-partner violence, which can be physical, psychological or sexual.^3^ One in three women worldwide will experience IPV or non-partner sexual violence in their lifetime, leading to poorer physical and mental health outcomes.^3^ A Swedish study found that 60% of respondents aged 15–19 had experienced some form of IPV.^4^

Sexual risk behaviour, defined as unprotected sexual intercourse, increases the risk of adverse health outcomes such as unwanted pregnancy and sexually transmitted infections (STIs).^5^ Factors associated with sexual risk taking include substance use,^6^ early sexual initiation,^7^ multiple sexual partners^8^ and limited ability to negotiate safer sex.^9^ There is a strong association between sexual risk taking, poor SRH and exposure to multiple forms of violence, and lesbian, gay, bisexual and transgender youths being particularly at risk.^10^

In Sweden, SRH promotion for youth is carried out through schools and youth-friendly clinics. All schools must have school health services (SHS) covering medical, psychological and social well-being needs.^11^ SHS are required to offer regular preventive health assessments for students, which can include SRH, but standardised protocols are lacking.^11^

Young people often hesitate to discuss SRH with professionals and rarely disclose experiences of sexual violence.^12^ Healthcare providers also infrequently and inconsistently address SRH topics with young people.^13^ A recent study in Sweden demonstrated that the systematic use of the SEXual health Identification Tool (SEXIT), an assessment tool to identify youth with experiences of sexual risk taking and violence, facilitated important conversations about SRH in youth clinics.^14^ SEXIT is increasingly being used within SHS,^15^ though its use and feasibility in this context have not yet been evaluated.

### Aim

The aim of this study was to explore school health professionals’ (SHPs) experiences of implementing structured assessments of sexual health and experiences of violence among youth in Sweden using SEXIT.

## Methods

### Study design

**Figure 1:**
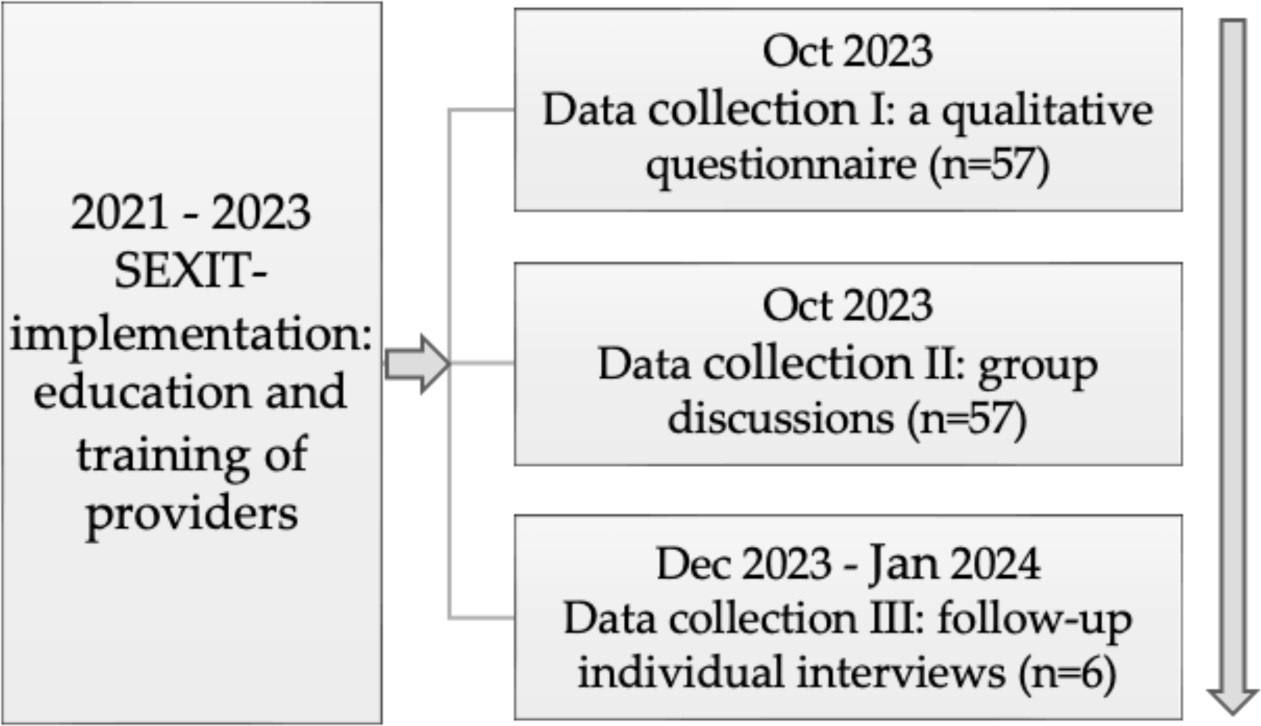
The qualitative sequential study design with a three-step data collection.

This study had a qualitative sequential design^16^ based on a qualitative questionnaire with open-ended questions, group discussions and individual interviews, to explore the experiences of SHPs trained in the SEXIT method in one county in Sweden (figure 1). The study was planned and reported according to the Consolidated Criteria for Reporting Qualitative Research Checklist.^17^

### The SEXual health Identification Tool

The SEXIT package includes staff training, a questionnaire and a guidance handbook to support conversations and risk assessments. SEXIT 3.0 is a 22-item questionnaire covering various topics, such as age, gender identity, sexual orientation, living situation, substance use, experiences of violence, sexual initiation, sexual partners, use of contraception and STI protection, previous STIs, unintended pregnancy, transactional sex, unwanted sexual experiences and sexual coercion.^15^

### Study population and setting

The informants were SHPs working in a Swedish county with a population of about 130 000, characterised by a diverse demographic and socioeconomic composition, with one-third of the population living in rural areas. Approximately 9000 students aged 13–19 are enrolled in grades 7–12 of lower and upper secondary schools. The implementation of SEXIT in the county is ongoing (2021–2024), aiming to train all SHPs. Participating professions included registered nurses, midwives, counsellors, social workers, psychologists and general practitioners, all trained in SEXIT, with varying levels of experience (0–2 years). Inclusion criteria consisted of SHPs who were trained in SEXIT and currently working within SHS in the county, while those who had not yet been trained were excluded.

### Questionnaire and interview topic guide

The qualitative questionnaire included five open-ended questions addressing the value of SEXIT interviews, potential outcomes, challenges, reasons for not yet initiating SEXIT assessments and perceptions of youths’ attitudes towards SEXIT (online supplemental file 1). The group discussions covered the same topics. The topic guide used for the follow-up interviews (online supplemental file 2) was informed by the findings from the questionnaire and group discussions, aiming to further explore and gain deeper insights.^17^

### Data collection

The data collection followed a qualitative sequential study design,^16^ as illustrated in figure 1. Steps I (a qualitative questionnaire survey) and II (group discussions) were conducted during a one-day workshop aimed at exchanging experiences related to the use of SEXIT and its implementation in the county. The workshop was organised by the county’s public health department at a conference facility with a capacity of 60 people in October 2023. At the time of data collection, 160 SHPs met the inclusion criteria and were invited to participate in the workshop on a first-come, first-served basis. Sixty SHPs participated, and all participants received an information letter and consent form 2 weeks prior. Three out of the 60 participants declined to participate in the study due to scheduling conflicts during the day. After a 30-minute briefing by the principal investigator (MM), the remaining 57 participants individually and anonymously completed the questionnaire (step I). Immediately afterward, they were split into smaller groups of 3–4 participants for 60-minute group discussions (step II), during which their discussions were documented using an online tool (Padlet).

During the workshop, six study participants were voluntarily recruited for individual semistructured online interviews (step III). This included three school nurses and three school counsellors from different SHS, representing both urban and rural areas. The interviews were conducted online by MH in December 2023 and January 2024, with SS acting as note-taker and using automatic transcription (Microsoft Teams). Each interview lasted 30–45 min, scheduled at a time chosen by the participant and conducted in a private setting.

### Data analysis

Qualitative content analysis was conducted according to Graneheim and Lundman.^18^ An inductive approach was taken to explore the SHPs’ experiences of using SEXIT, allowing for the discovery of new concepts.^19^ The data from steps I and II were read repeatedly to extract meaning units, which were then condensed and labelled with codes. MH and SS coded the data concurrently and separately to ensure accuracy. The codes were compared, grouped into subcategories, and then into categories through reflection and discussion among MH, SS and MM. Following the analysis and comparison of the two data sources, it became evident that the insights from the group discussions (step II) aligned with the results of the questionnaire (step I), with no new codes emerging, suggesting that saturation had been reached.^20^

The data from step III were analysed using a deductive approach as the focus shifted to verifying and deepening the understanding of the previous findings.^19^ The data were coded and grouped according to the subcategories identified in steps I and II by MH, and this process was verified by MM. The predetermined subcategories were well represented in the data collected from the individual interviews (step III), suggesting saturation.^20^ Finally, two themes emerged through latent content analysis, unifying the content within the categories.^18^ Four informants validated the results. The analysis was conducted in Swedish, and the main findings, along with illustrative quotes, were translated into English.

### Patient and public involvement

SEXIT was developed and tested in collaboration with youths.^21^

## Results

The analysis revealed 17 subcategories, eight categories and two themes—SEXIT aids providers in acting as educators promoting sexual health and freedom from violence, while a lack of supportive leadership and a unified approach hinders effective implementation (table 1).

**Table 1:** Overview of themes supported by categories and subcategories, illustrating SHPs’ experiences of the implementation of SEXIT

SEXIT aids providers in acting as educators promoting sexual health and freedom from violence	Lack of supportive leadership and a unified approach hinders effective implementation
Reduces stigma	Lack of time and resources
normalises conversations about sensitive topics provides an opportunity for education on sexual health and violence positive reception among the youth	time-consuming or a time-saver? an additional burden
Nuanced discussions on violence	Vulnerable groups need increased attention
puts online violence in the spotlight identifies the perpetrator of violence	language and cultural barriers youth with disabilities are disadvantaged
Identifies poor sexual health and risk-taking behaviour	Need for clearer guidelines
enables early identification of risk factors ensures consistency and quality in the preventative work	uncertainties about roles and responsibilities lack of consensus regarding appropriate age for introduction
Facilitates providing youth with support	Collaboration with external organisations required
promotes relationship building creates a thorough basis for referrals	efficient referral pathways need to be established collaboration between the schools and community services is crucial

SEXIT, SEXual health Identification Tool; SHP, school health professional

### SEXIT aids providers in acting as educators promoting sexual health and freedom from violence

SEXIT promoted education on SRH and violence (table 1), requiring SHPs to explain terms such as “sexual orientation” and various forms of violence. It also provided a platform for discussing sensitive topics, helping informants establish trustful relationships with youth, which is essential for identifying those in need of support.

Many youths struggle to understand the questions in the SEXIT questionnaire, few are aware of the different concepts and definitions and do not know if they have been victims of violence or not. Hopefully, their tick in the “I don’t know”—box becomes an “aha” moment for them that leads to self-reflection and continued conversations about what violence actually is. (informant 37)

### Reduces stigma

The detailed descriptions in SEXIT helped explain terms and normalise conversations about sensitive topics such as sexual initiation, unintended pregnancy, contraception, STIs and various forms of violence. This approach reduced the stigma associated with these topics among both students and professionals. Informants noted that the routine use of SEXIT ensured no student felt targeted by specific questions. SHS were described as safe spaces for youth to discuss personal concerns, facilitating the implementation of SEXIT. Although some youth seemed embarrassed, most took the conversations seriously and did not view them as a ‘big deal’. Participation in SEXIT assessments is voluntary but reportedly high.

I find it [SEXIT] most valuable with young men. In general, they struggle more to talk about these issues and often present with physical complaints to me as a doctor. (informant 4)

It [SEXIT] does not require much extra time during the regular health assessment, and it becomes a natural opportunity to bring up these topics with everyone, that way no one feels singled out. (informant 1)

I have rarely experienced that youth decline to answer all or even some questions. Some feel uncomfortable, but most of them do not seem to think that it is strange to be asked. (informant 37)

### Nuanced discussions on violence

SEXIT helped informants define ‘violence’ and broaden discussions about various forms of violence with youth. For younger students (12–13 years), conversations focused more on relationships with friends and family and experiences of violence, rather than SRH-related issues. The detailed questions about violence highlighted the normalisation of online bullying and sexualised violence. SEXIT also facilitated the identification of perpetrators of violence, marking a shift from a previous focus solely on victimisation. Informants were surprised by the youth’s honesty and had identified not only victims but also perpetrators. However, there was uncertainty about where to refer young perpetrators and what support is available to them.

The SEXIT questions are phrased in a way I would not otherwise phrase questions about violence myself. […] In my experience, the detailed questions make the issue of experience of violence more comprehensible to the youth. (informant 38)

Often good conversations around “dickpics”, “nudes”, etc. (informant 48)

Enables the perspective of the perpetrator, that it is possible to talk about it. (informant 33)

### Identifies poor sexual health and risk-taking behaviour

SEXIT was described as a valuable tool for identifying risk factors and ongoing SRH issues, as well as exposure to violence. It was emphasised that SEXIT complemented the regular health assessments, which often lack specific inquiries in these areas. Additionally, it also helped identify underlying causes of problematic behaviour and absenteeism, enhancing the preventive work within SHS. The routine use of SEXIT improved the consistency and quality of health consultations, as all students were asked the same questions. Informants valued the questionnaire as a conversation starter, easing the pressure on youth to initiate discussions.

SEXIT often provides useful information—about previous/ongoing exposure to violence, risk-taking, alcohol/drugs. It leads to a conversation about things that the youth perhaps did not present for, but that is of great importance to their health. (informant 56)

A helpful way to approach this area and introduce this conversation. A good complement to the regular health assessment questionnaire, where these questions are not asked so specifically. (informant 49)

Using SEXIT (along with the handbook), I find that it is easier for both the students to answer the questions and for me to ask them (compared to a “regular” consultation). (informant 13)

### Facilitates providing youth with support

The use of SEXIT, along with its recurrent presence within the school and community-based youth health services, was perceived as enhancing relationship building between youths and professionals. By consistently being exposed to SEXIT, youth became aware of where to seek help when they had issues or questions, even if they were not ready to discuss them during their initial visit. Informants also noted that when more specific questions were asked, the responses tended to be more detailed, facilitating the compilation of clearer and more comprehensive reports and referrals.

The value lies in that it [SEXIT] becomes a screening of everyone […] to be asked multiple times, then maybe the third time the student “dares” to answer “yes” for example. If the students wish to talk further about any question, they know that “I can talk to the school nurse about these questions”—when they feel ready. (informant 57)

I find it [SEXIT] very valuable. We meet the students in their everyday life and our availability must be utilised. (informant 6)

### Lack of supportive leadership and a unified approach hinders effective implementation

A successful full-scale implementation of SEXIT depends on supportive leadership and a unified approach from all organisations involved (table 1). This includes addressing key factors such as resource allocation, clear guidelines, increased attention on vulnerable groups and collaborative efforts with external partners.

I have started using SEXIT, but the biggest challenges are time and to know what to do with the potential issues identified. It is important that this is clear before implementation. (informant 55)

### Lack of time and resources

The lack of time and resources were the main issues hindering or slowing SEXIT implementation within some organisations. Informants who had not yet started using SEXIT viewed it as just another form to fill out. Some school health nurses hesitated to extend the already lengthy regular health assessment and were concerned about managing follow-up appointments without additional resources, such as protected time or more colleagues. Conversely, informants who had been using SEXIT for some time described it as a time saver, noting that the structured questionnaire facilitated the efficient and sensitive asking of difficult questions, helping them identify issues more quickly.

We are positive within the organisation to introducing SEXIT, but unfortunately are given no further resources to do the work. It just becomes an added burden on us. (informant 52)

Unfortunately, there is not enough time or resources to use SEXIT, even though it would be desirable, but the number of students per school nurse is too high. (informant 24)

SEXIT is an anticipated complement to the questionnaire we use at the regular health assessment in grade 7. It takes about 5-20 minutes to complete depending on the student and is a good basis for conversation. It has been a valuable help to me. (informant 17)

It [the use of SEXIT] has led to deeper conversations and questions around topics that would have been harder to get to, or that had required more time to get to, without it. (informant 31)

### Vulnerable groups need increased attention

Youth with limited understanding of the Swedish language and those with intellectual disabilities were identified as facing significant challenges in using SEXIT. Informants were concerned that language and cultural barriers could lead to misinterpretation and under-reporting of issues with some youth of immigrant background. Additionally, most informants completely avoided using SEXIT with youth who had intellectual disabilities, assuming that the disability would hinder their full comprehension of the questions. The informants expressed a need for tailored interventions and adaptations, such as simplified language, culturally relevant content and alternative communication methods, to ensure all youth can access and benefit from SEXIT.

Consultations with students who do not speak Swedish take longer and sometimes I have felt uncertain whether these students could fully express what they wanted to say. (informant 17)

It [SEXIT] can rarely be used with students with intellectual disabilities. (informant 30)

### Need for clearer guidelines

The implementation process of SEXIT varied, with some workplaces initiating it at the management level and others at the individual staff level. Concerns were raised about inconsistencies in addressing SRH issues, with some colleagues hesitant to ask SEXIT questions. In some schools, nurses incorporated SEXIT in the regular health assessments, while in others, counsellors managed it. Some workplaces adopted a collaborative approach involving multiple professions. Opinions differed on which profession should use SEXIT, but most agreed that the key was having someone comfortable discussing these topics.

I still feel quite unsure on the [SEXIT] material […] there are still no clear guidelines regarding SEXIT within my organisation, I think that would make it easier. (informant 3)

It is still unclear which profession within the SHS that should use it [SEXIT], and when. (informant 1)

Early introduction of SEXIT was seen as beneficial for normalising discussions around SRH and violence, as well as equipping youth with preventive knowledge. Although younger students often required more explanation and rarely provided substantive responses, some viewed this as an opportunity to raise awareness and educate.

Many youths struggle to understand some of the concepts/questions—but then we talk about them and explain. As I said, it seems like they are “too young” in grade 7 but also not, because it opens up for discussions. Someone who has been victimised is never too young to get the question and will often need to be asked multiple times before they dare to tell. So, a follow up in grade 9 might be good. (informant 18)

### Collaboration with external organisations required

For successful SEXIT implementation and equitable support for youth, informants emphasised the need for strong collaboration between schools and external organisations, such as the police, social services, youth clinics and psychiatry services. There was considerable uncertainty about where to report or refer cases, particularly those involving young perpetrators of violence, which made some informants uncomfortable asking questions. The informants requested streamlined referral pathways to enable SHPs to confidently address and act on youth responses to SEXIT.

Clear information to the youth that they will be offered to fill out SEXIT throughout all of lower and upper secondary school, as well as at visits at youth psychiatry services and youth friendly health clinics, as a routine… would probably result in better outcomes. (informant 18)

I rarely have the solutions to the issues that might be identified but I can refer, which might lead to feelings of frustration and powerlessness. (informant 5)

## Discussion

SEXIT promoted educational opportunities that helped reduce stigma, normalise conversations about sensitive topics, and identify students in need of support. However, supportive leadership and a unified approach are required for further implementation to clarify roles, responsibilities and referral pathways, as well as to ensure equitable SHS.

The systematic use of a questionnaire as basis for conversation with all youth was perceived as increasing the consistency and quality of the preventive work within SHS. Previous studies on routine assessments of sexual health, alcohol consumption and violence victimisation have also emphasised the importance of systematic approaches to assess health risks among youth.^22 23^ SHPs have a unique opportunity to educate and promote SRH to all youth due to their presence in students’ daily lives.^24^ Implementing preventive measures, such as the routine use of SEXIT, provides an opportunity to reduce poor SRH at an early age. However, our findings suggest that without leadership support for these routines, providers’ personal beliefs, values and attitudes related to sexuality may affect how and with whom SRH questions are raised.

Previous research recommends intervening during early adolescence (around age 13), when romantic relationships begin to emerge and violent behaviours have not yet surfaced for most individuals.^25^ Some studies suggest starting even earlier to address predictors of dating violence, such as bullying and sexual harassment, which are common in this age group.^26 27^ Our findings indicate that routine use of SEXIT within SHS provides a natural opportunity to address SRH topics early, often before sexual initiation, helping to shape healthy sexual relationships throughout the lifespan. However, despite caregivers and adolescents recognising the importance of discussing SRH topics with healthcare providers, these discussions often do not occur during preventive health visits, resulting in missed critical opportunities for screening, education and guidance.^22 28^

Students with an immigrant background and/or intellectual disabilities were described as doubly vulnerable, facing a higher risk of poor SRH outcomes and violence, while also being harder to detect and support. Youths from immigrant backgrounds encounter language and sociocultural barriers that may hinder their access to SRH services, often lacking knowledge about where to seek help.^29 30^ Those with intellectual disabilities tend to engage in unsafe sex more often than their peers and have significant unmet needs for SRH services.^31 32^ Tailored interventions and adaptations of SEXIT are essential to ensure that all youths can access the necessary services. Currently, SEXIT has been translated into several languages,^15^ but there appears to be limited awareness and use of these versions within SHS.

### Methodological considerations

The study had limitations due to its restricted geographical area. However, several factors enhanced its trustworthiness. Purposive sampling created a diverse sample in terms of age, occupation and work experience, thereby strengthening the credibility of the results.^18^ This diversity was advantageous for exploring different perspectives, although the impact of hierarchy on group discussions could not be entirely excluded. The qualitative sequential study design was a significant asset, allowing for a comprehensive understanding and cross-checking of findings through methodological triangulation.^16 33^ The group discussions (step II) enabled informants to share and clarify their experiences collectively, while the individual interviews (step III) confirmed the findings.^17^ The short data collection period minimised the risk of external factors that could affect the outcome, enhancing dependability.^18^

To ensure transferability, the study context, informant characteristics, data collection and analysis processes were clearly described. Additionally, quotes were presented to illustrate the connection between the data and findings, further strengthening trustworthiness.^18^

### Implications of results and future research

This study builds on previous research on the use of SEXIT within youth-friendly clinics^14^ by evaluating its application within SHS. The findings are valuable for policy development and for the continued implementation of SEXIT on a larger scale in various settings. SEXIT provides an opportunity to identify perpetrators of violence; however, referral pathways for these individuals need to be established. Further research is required on the use of SEXIT among youth from immigrant backgrounds and/or intellectual disabilities.

## Conclusions

SEXIT has the potential to provide an equitable platform for discussing sensitive topics such as sexual risk taking and violence, fostering a deeper connection between youths and professionals. However, SEXIT is not yet routinely used in all SHS, highlighting the need for supportive leadership and a unified approach among organisations. Addressing sexual risk taking and violence through SEXIT is considered a priority to help youths make informed SRH choices, achieve healthy relationships and attain freedom from violence.

## supplementary material

10.1136/bmjph-2024-001667 online supplemental file 1

10.1136/bmjph-2024-001667 online supplemental file 2

## Data Availability

No data are available.
