# Supplementary material for: Experiences of school health professionals in implementing structured assessments of sexual health and experiences of violence among youth in Sweden using the SEXual health Identification Tool (SEXIT): a qualitative sequential study
Source: BMJ Public Health. 2024 Nov 26;2(2):e001667. doi: 10.1136/bmjph-2024-001667 (PMC11816194; doi:10.1136/bmjph-2024-001667)
Supplement: online supplemental file 1 [file bmjph-2-2-s001.pdf]

Early identification of school youth at risk to provide them with support that promotes sexual health and freedom from violence

Questions for Participants at the Experience Exchange Day Regarding the SEXIT Work, Autumn 2023

1. How do you perceive the value of conducting SEXIT conversations?

(… and please describe this in relation to other responsibilities that you have)

2. How do you perceive the youth's experiences of the conversation?

(… any differences based on gender and sexual orientation)

3. What can the conversations lead to?

(… and please provide examples without personal information about students)

4. What obstacles/difficulties do you encounter in the conversation?

(… any differences depending on gender and sexual orientation)

5. If you have not yet started using SEXIT, what are the reasons/challenges that need to be overcome?

(… categorise the level at which you perceive the challenges exist, and how you view the future/potential solutions)
