# Supplementary material for: Experiences of school health professionals in implementing structured assessments of sexual health and experiences of violence among youth in Sweden using the SEXual health Identification Tool (SEXIT): a qualitative sequential study
Source: BMJ Public Health. 2024 Nov 26;2(2):e001667. doi: 10.1136/bmjph-2024-001667 (PMC11816194; doi:10.1136/bmjph-2024-001667)
Supplement: online supplemental file 2 [file bmjph-2-2-s002.pdf]

Early identification of school youth at risk to provide them with support that promotes sexual health and freedom from violence

Interview Guide – In-Depth Individual Semi-Structured Interviews, Autumn 2023

- How do you use SEXIT?

(when/in what context? with whom?)

- Follow-up question if not using it as a screening tool with everyone – how do you choose/whom do you target?

- How do you think SEXIT conversations should be conducted, and by whom?

(which profession and when/in what context, e.g. planned as a screening of all students or only on indication)

- When do you think is an appropriate grade/age to introduce SEXIT?

(please justify why, including advantages/disadvantages)

- "Not having time to build a relationship before difficult questions are asked" – do you perceive this as a problem with SEXIT, or can SEXIT, conversely, bridge this gap?

(why/in what way?)

- What value do you see in using SEXIT?

(e.g. is there value even when there isn't a "positive outcome"?)

- What obstacles do you experience in conducting the conversation (if any)?

- Are there any problems with SEXIT?

(e.g. difficult concepts or poorly phrased/problematic questions)

- What support is provided from the leadership level in your organisation/at your workplace to implement/conduct SEXIT?

- How do you deal with "positive outcomes"?

(is it clear to you what actions you need to take? e.g. how to refer someone and where to)
